# Emotion and Belief

**Published:** 1917-03-31

**Authors:** 


### EMOTION AND BELIEF.

"We are not," says Bacon, "to expect the discovery of things useful in common life . . . from abstract philosophies," but we are to rely rather upon "a sagacious experience and general knowledge of nature." Such a method requires, however, the expenditure of time and energy in acquiring knowledge by means of observation and experiment, and it is little likely, therefore, to be much in favour with those who prefer to have their conclusions ready-made. For the majority of people it is natural to take the path of least resistance. It has been so from time immemorial, and everything goes to prove that so it will continue to be for long days to come. Each generation starts afresh only in a limited way in so far as knowledge is concerned. There may be an increasing potentiality for right-thinking: but in the majority of cases the environment is not so altered as to make this apparent. There is, too, an intense conservatism in nature which tends to retain in, for example, the human body structures which have outlasted their useful function. They are reproduced in a dwindling form from generation to generation, becoming at length, before they are eliminated, actually noxious. This conservatism is exemplified strikingly in cerebral function. The brains of different individuals tend to respond to certain stimuli, while they remain practically uninfluenced by others. Where there is a great change in the environment—and in this term is comprised every influence brought to bear upon the body—quite remarkable differences may result in the individual. But such changes are not so frequent as is commonly imagined: and the individuals of one generation vary little, therefore, from those of another.

For those who hold fast to belief in an abstract mind and a consequent freedom of the will, it is difficult to understand why mankind has retained, almost unimpaired, characteristics which were present in a very primitive state of society; nor is it easy for them to comprehend why after so many centuries high ideals of love for one's neighbour, charity, and peace have had so little influence upon the human character. When, however, it is realised that changes in that direction are only brought about by a slow process of evolution—the matter should become at once more easily comprehended. Those functions of the brain which are called psychologically the higher mental attributes have evolved only recently in our history; and they are dependent upon the integrity of cells which are, up to the present time, probably the least stable parts of the nervous system. Consciousness, in the ordinary acceptation of the term, may be included in the same category. Yet it is upon these comparatively unstable nerve-cells that we depend for our interpretation of the messages which are brought to them from the organs of sense. Various substances—such, for example, as alcohol, morphia, and haschish—interfere with their function and render them incapable of accurately interpreting stimuli. Very similar results are brought about by circulatory changes such as occur during emotional stress; and it is not, therefore, wise to accept readily as facts the statements of people who are under the influence of strong emotions. They may believe that they speak truthfully; but strength of belief is not a reliable criterion of accuracy.

No one is more convinced of the reliability of his testimony than is the insane person: to him his illusions and hallucinations are indubitable evidence of objective realities. No definite dividing line can, however, be drawn between sanity and certain forms of insanity. It seems, therefore, somewhat hazardous to base systems of belief upon the testimony of people whose nervous organisation is either hereditarily defective or has been rendered unstable either by excessive stimulation or by some noxious substance in the blood-stream.

Such matters as these are, for the most part, scornfully set aside by those people who, with an ardent will-to-believe, accept any evidence which gives support to their doctrine; or, on the other hand, they are perverted to serve the same purpose. Very frequently, however, they are quite unknown even by those who are most dogmatic. The mere mention of such well-known phenomena as illusions and hallucinations is sufficient to provoke the indignation of those who resent any attempt to explain scientifically what is at the basis of their beliefs. Yet a proper understanding even of such matters as these would prevent many from being led astray and from cherishing hopes that can but be disappointed. At the present time the will-to-believe is strong—as it always is, indeed, when the emotions are predominant. Men who are eminent in certain spheres of knowledge allow themselves to be convinced of what they believe to be truth by evidence which, in their own special lines of research, they would reject as valueless. With very little—or with no—knowledge of the functions of the nervous system they are yet prepared to accept as supernatural—or extranatural—phenomena which are the commonplaces of psychiatry. The general public are not, however, able to judge calmly of their merits, and consequently they attach unmerited importance to their statements. They do not realise that "a person truly eminent in one department of knowledge to which he has applied his mind diligently and systematically (mathematicians especially) is notably sometimes extremely credulous—almost as it were hypnotised—in relation to matters of which he has not been wont to think, or not taught himself to think seriously."

Such considerations need not, however, make us despair of the progress of rational conceptions. Although we may try to rid ourselves of the influence of our environment by concentrating our attention upon subjective states, yet, however slowly, the stimuli from without mould our nervous systems and render them more capable of perceiving and comprehending with increasing accuracy. Until, however, the process of evolution has made still further advance there will be plenty of plastic material whereof the teachers of mysticism in all its phases may fashion converts.

